# Cardiac magnetic resonance imaging can be performed without the use of anesthesia in patients 7-10 years of age with child life support and MRI video goggles

**DOI:** 10.1186/1532-429X-18-S1-O120

**Published:** 2016-01-27

**Authors:** Denna Jung, John Wood, Karen Gloer, Gabriela I Hernandez, Susanne Matich, Fariba Goodarzian, Samuel Yanofsky, Patrick A Ross, Jon Detterich

**Affiliations:** Children's Hospital Los Angeles, Los Angeles, CA USA

## Background

Cardiac magnetic resonance imaging (CMR) is utilized as both a primary and adjunctive imaging modality for the diagnosis and evaluation of congenital heart disease, cardiomyopathy and characterization of myocardial iron loading. Adequate image quality and accurate diagnosis is dependent on the patient lying still; however, CMR exams can be long, requiring children to lie still for periods of time up to 1.5hours. In order to limit motion in children, anesthesia services are often required; and prior to August 2012 our institution did not offer awake CMR prior to 10 years of age unless extenuating circumstances exist. After August 2012, our CMR team instituted a change in policy to attempt CMR without anesthesia in patients less than 10 years of age if the educational tools utilized by child life were successful.

The aim of this study was to determine whether our institution can decrease anesthesia utilization, safely, for CMR in children age 7-10 years of age while using child life specialists prior to CMR and video goggles during CMR.

## Methods

This retrospective, cohort study included patients 7-10 years of age undergoing CMR imaging over two periods of time, August 2010-August 2012 (Era 1) and August 2013-August 2015 (Era 2). Era 1 was prior to an institutional policy change to attempt CMR exams without anesthesia in subjects less than 10 years of age. Era 2 had CMR exams performed following implementation of a program to perform CMR without anesthesia in the same age range. Child life specialists and video goggles were being employed in our Radiology department throughout both eras. However, the goal during Era 2 was to assess the suitability of attempting a CMR exam awake, in patients scheduled for CMR under anesthesia. Categorical analysis was used to determine differences between Eras.

## Results

**Figure 1:**
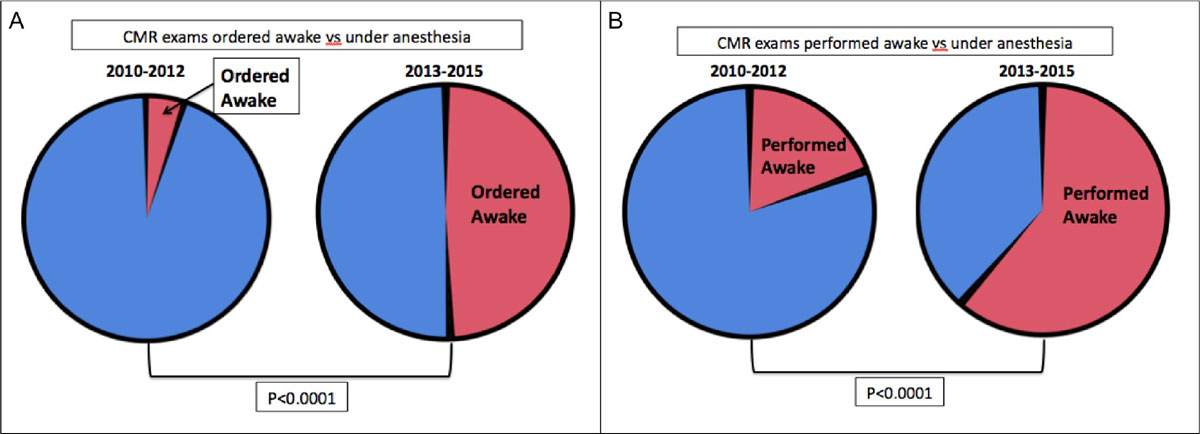
Figure 1

There were 269 patients who underwent CMR over both periods studied, Era 1 had 102 patients and Era 2 had 167 patients. Anesthesia was ordered in 97% (Figure 1A) but only utilized in 80% of scans for Era 1 (Figure 1B). Anesthesia was ordered in 61% but only utilized in 39% of scans for Era 2 (Figure 1A and 1B), p < 0.0001). For both Eras, there was no difference in the number of patients who were scheduled for anesthesia but attempted CMR awake(p = 0.13). Furthermore there was no difference in the number of patients who failed an attempted CMR without anesthesia in Era 1 vs Era 2(p = 0.40).

## Conclusions

CMR can be successfully performed in patients between 7 and 10 years of age without anesthesia, while utilizing child life educational tools and video-assisted CMR. This strategy decreases the need for anesthesia without increasing scan failure rate.

**Figure 2:**
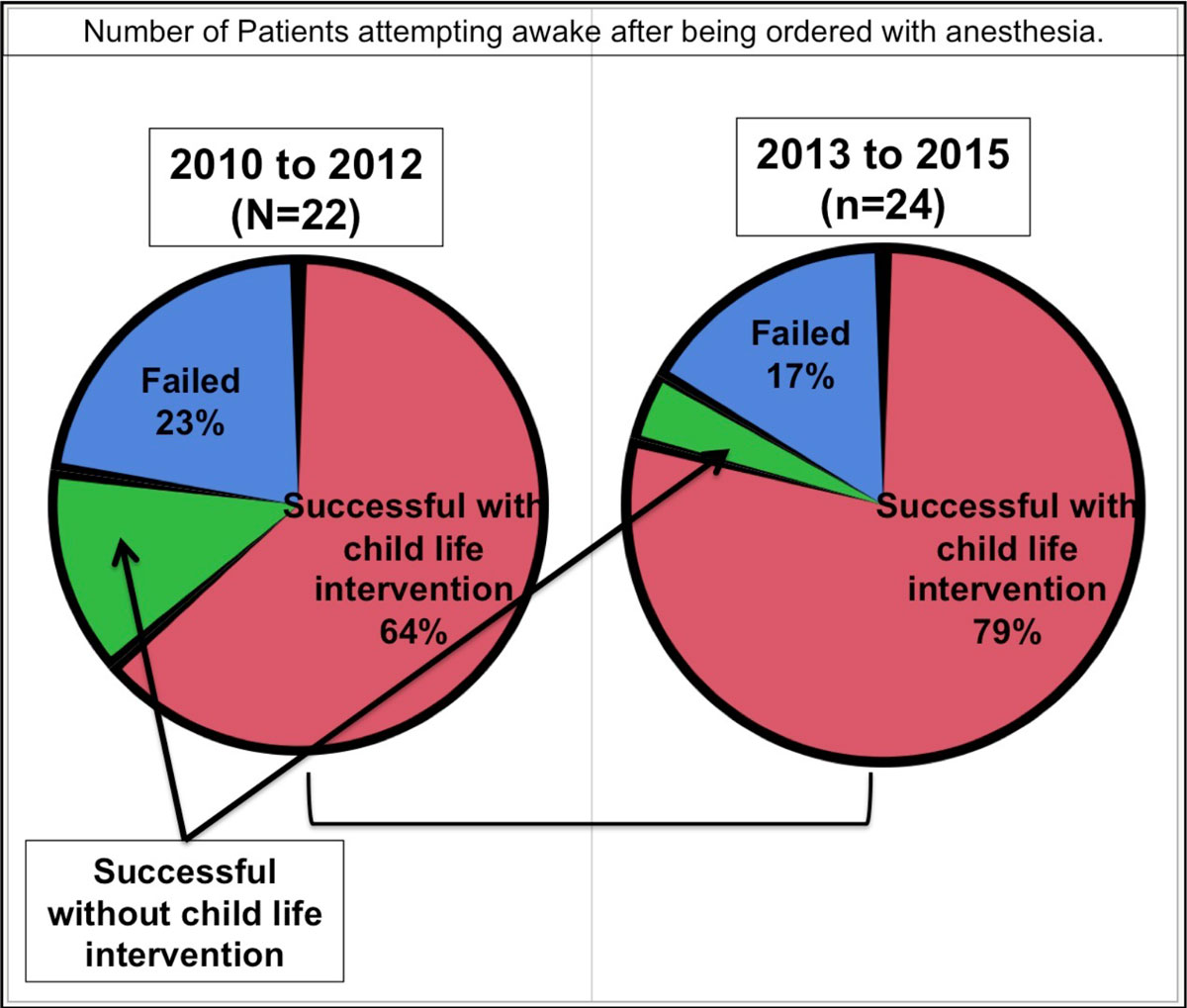
Figure 2

